# Comparative Studies on the Anti-Inflammatory and Apoptotic Activities of Four Greek Essential Oils: Involvement in the Regulation of NF-κΒ and Steroid Receptor Signaling

**DOI:** 10.3390/life13071534

**Published:** 2023-07-10

**Authors:** Achilleas Georgantopoulos, Athanasios Vougioukas, Foteini D. Kalousi, Ioannis Tsialtas, Anna-Maria G. Psarra

**Affiliations:** Department of Biochemistry and Biotechnology, University of Thessaly, Biopolis, 41500 Larissa, Greece; ageorgant@uth.gr (A.G.); atvougioukas@uth.gr (A.V.); fokalous@uth.gr (F.D.K.); tsialtasj@gmail.com (I.T.)

**Keywords:** essential oils, *Melissa officinalis*, oregano, lavender, Chios Mastic (Mastiha), glucocorticoid receptor, anti-inflammatory actions, apoptosis, anti-hyperglycemic actions

## Abstract

Essential oils (EOs) are well-known for their anti-fungal, anti-microbial, anti-inflammatory and relaxing activities. Steroid hormones, especially glucocorticoids, are also well-known for their anti-inflammatory activities and control of the hypothalamus–pituitary–adrenal (HPA) axis and glucose homeostasis. The biological activities of glucocorticoids render them the most widely prescribed anti-inflammatory drugs, despite their adverse side effects. In this study, comparative studies of the anti-inflammatory activities and interference with glucocorticoids receptor (GR) and estrogen receptor (ER) signaling of EOs from Greek Oregano, *Melissa officinalis*, Lavender and from the Chios Mastic, produced from the Greek endemic mastic tree, were performed in Human Embryonic Kidney 293 (HEK-293) cells. Chios Mastic (Mastiha) and oregano EOs exhibited the highest anti-inflammatory activities. The former showed a reduction in both NF-κB activity and protein levels. Mastic essential oil also caused a reduction in GR protein levels that may compensate for its boosting effect on dexamethasone (DEX)-induced GR transcriptional activation, ending up in no induction of the gluconeogenic phoshoenolpyruvate carboxykinase (PEPCK) protein levels that constitute the GR target. Oregano, *Melissa officinalis* and lavender EOs caused the suppression of the transcriptional activation of GR. Furthermore, the most active EO, that taken from *Melissa officinalis*, showed a reduction in both GR and PEPCK protein levels. Thus, the anti-inflammatory and anti-gluconeogenic activities of the EOs were uncovered, possibly via the regulation of GR signaling. Moreover, cytotoxic actions of *Melissa officinalis* and lavender EOs via the induction of mitochondrial-dependent apoptosis were revealed. Our results highlight these essentials oils’ anti-inflammatory and apoptotic actions in relation to their implication on the regulation of steroid hormones’ actions, uncovering their potential use in steroid therapy, with many applications in pharmaceutical and health industries as anti-cancer, anti-hyperglycemic and anti-inflammatory supplements.

## 1. Introduction

Essential oils are extremely complex mixtures of volatile compounds particularly abundant in aromatic plants, which are mainly secondary metabolites, such as terpenoids and terpenes, which are biogenerated by the mevalonate pathway [1]. Aromatic monoterpenes and sesquiterpenes are the most abundant ones [2,3]. Essential oils are isolated from various anatomical parts of these plants and are produced via extraction, compression or/and distillation methods. They have been used for many years in different industrial sectors, such as cosmetics and personal care products, and are also widely used as food flavoring additives. The anti-microbial and anti-fungal effects of certain essential oils are well-known. Nowadays, an increasing number of studies have uncovered essential oils’ anti-inflammatory, anti-oxidant and/or anti-cancer activities, which are attributed to their chemical composition of individual compounds or compounds in the mixture [4,5].

Essential oils’ compounds, such as terpenes and triterpenes, are steroid-like compounds that could possibly interfere with steroid receptor signaling. Glucocorticoid and estrogen receptors are steroid receptors with remarkable anti-inflammatory activities, mainly via their interaction with the inflammatory factor ΝF-κB and the suppression of its activity [6,7,8,9,10,11]. In this study, four essential oils from three Greek aromatic plants, namely Oregano, *Melissa officinalis* and Lavender cultured in Thessaly, a Greek agricultural mainland region, and Chios Mastic essential oil from the resin of the endemic *Pistacia. Lentiscus L. var. Chia*, cultured exclusively in the southern part of the Greek island of Chios, were evaluated for their anti-inflammatory actions with respect to their interference with steroid receptor signaling. Particularly, the investigation of the potential dissociative activities of these essential oils on the steroid receptors’ actions that lead to the suppression of TNF-α-induced NF-κB activation (and thus anti-inflammatory activities), with no or limited effects on the steroid receptors’ transactivation (and thus possible gluconeogenic activities, as regards the glucocorticoid receptor) raised our interest [10,12]. Moreover, based on steroid receptors’ crucial role in the regulation of energy metabolism and apoptosis, acting as transcription factors and direct or indirect regulators of energy metabolism- and apoptosis-related gene expression [13,14,15,16], the possible interference of the essential oils with the steroid hormones, especially the glucocorticoids signaling pathway, is an interesting issue to be explored.

Chios Mastic oil is produced through the distillation of Chios Mastic gum, the air-dried resinous substance from *Pistacia Lentiscus L. var. Chia* (mastic tree). The mastic tree is native to the Mediterranean region and is principally cultivated on the southern part of the Greek island of Chios. Compounds such as a-pinene and β-myrcene are predominantly found in Mastic oil [17]. Studies have shown that Chios Mastic οil may exhibit anti-oxidant, anti-lipidic and anti-cancer activities [18,19,20,21]. In addition, accumulating evidence has proven the anti-inflammatory actions of essential oil from the mastic tree, via inflammatory factors, including Interleukin-6 (IL-6) and Tumor Necrosis factor-alpha (TNF-α) reduction [22].

Similarly, *Melissa officinalis* essential oil has been shown to exhibit strong anti-bacterial and anti-fungal activities. In particular, it has been shown to inhibit the growth of several species of Gram^+^ and Gram^−^ bacteria, as well as fungi [23]. Moreover, beyond its anti-microbial actions, many other biological actions and therapeutic properties of *Melissa officinalis* essential oil have been uncovered, such as its anti-cancer activity, highlighting its potential pharmaceutical applications [24,25]. Additionally, possible anti-hyperglycemic and neuroprotective actions of *Melissa officinalis* essential oil have been proposed, attributed to its effect on the reduction in PEPCK and glucose 6-phosphatase (G6Pase) [26] and hypoxia-inducible factor 1 (HIF-1) [27], respectively.

As regards lavender essential oil, it has been used for many decades in the field of aromatherapy due to its positive effect on increasing the duration of sleep, its muscle relaxant properties as well as its anxiolytic and sedative effects [28,29]. In addition, recent studies have shown lavender essential oil’s positive effects on wound healing [30]. Moreover, there are both in vitro and in vivo studies demonstrating the pro-apoptotic and anti-proliferative effects of lavender essential oil [31].

Oregano oil, in most cases, is prepared by drying the leaves and stems of the plants. Then, through steam distillation, a concentrated essential oil is produced. The chemical composition and content of oregano oil depend on a variety of factors, such as cultivation and growth conditions [32]. Oregano oil is proposed to exhibit anti-microbial, anti-inflammatory and anti-cancer activities, which are likely attributed to its composition of terpenoids [33,34,35]. In the same context, an increasing number of studies indicate its dose-dependent anti-proliferative properties [34] and its effect on the induction of apoptosis [36,37]. More interestingly, the anti-inflammatory effects of oregano essential oil have been suggested, in line with its chemical composition, enriched in carvacrol and thymol, which are involved in the reduction in reactive oxygen species (ROS) and nitric oxide levels, crucial mediators of inflammation [36].

Thus, many studies in the literature uncovered the plethora of the biological activities of essential oils from oregano, *Melissa officinalis*, lavender, and Chios Mastic. Nevertheless, none or a limited number of them have focused on the interference of these essential oils with steroid receptor signaling and, more precisely, on the characterization of the biochemical mechanisms and the possible biological impact of this action.

In this comparative study, the pro-apoptotic, anti-inflammatory and possible anti-hyperglycemic actions of four Greek essential oils, namely Chios Mastic Oil, *Melissa Officinalis* essential oil, oregano essential oil and lavender essential oil were evaluated. Emphasis was given to the investigation of the essential oils’ potential involvement in the regulation of the inflammatory factor NF-κΒ and the glucocorticoid and estrogen receptors (ERs) signaling pathway. To this purpose, comparative studies on the effect of the four essential oils on cell viability, interference with Estrogen receptor alpha (ERα), Estrogen receptor beta (ERβ), Glucocorticoid receptor and NF-κB signaling in the human embryonic kidney HEK-293 cell line were performed, applying MTT, luciferase assays, and Western blot analysis. 

## 2. Materials and Methods

### 2.1. Chemicals

Dulbecco’s Modified Eagle Medium (DMEM), Trypsin, and Fetal Bovine Serum (FBS) were obtained from Thermo Fisher Scientific (Thermo Fisher Scientific GmbH, Basel, Switzerland). Molecular protein weight marker was purchased from Proteintech (Rosemont, IL, USA, North America). TNF-α was purchased from PeproTech EC, Ltd. (London, UK). Cocktail protease inhibitors were purchased from Roche (Mannheim, Germany). Reporter lysis 5× buffer and luciferin were purchased from Promega Coorporation (Madison, WI, USA). DEX and Estradiol (E2) were obtained from Sigma-Aldrich (St. Louis, MO, USA). The Greek Oregano, Lavender, and *Melissa officinalis* essential oils were provided by Tharros Company Aromatic Plants Products, Larissa Greece, and the essential oil from Chios Mastic tree was provided by the Chios Mastic Growers Association and Mastiha Shop. Essential oils used in the study were 100% natural products without further impurities, as indicated by the chemical analysis, provided by the companies (Supplementary Data S1). For the biochemical assessment, essential oils were diluted in dimethyl sulfoxide (DMSO) (1 V/1 V) and were subsequently added to culture media in the indicated dilutions. 

### 2.2. Cell Culture

HEK-293 cell line was obtained from the American type culture collection (ATCC). HEK-293 non-cancerous cells were used in this study due to their high efficiency in transfection experiments, their considerable endogenous GR and NF-κΒ levels, and the extent of their use in receptor signaling studies and drug testing. Cells were cultured in 4.5 g/L glucose DMEM with phenol red, 10% *v*/*v* FBS, 2 mM L-glutamine and 100 units/mL penicillin–streptomycin at a temperature of 37 °C and 5% CO_2_. For hormone depletion, 48 h before the treatment, cells were cultured in 4.5 g/L glucose DMEM without phenol red, 10% *v*/*v* charcoal–dextran stripped FBS, 2 mM L-glutamine and 100 units/mL penicillin–streptomycin. 

### 2.3. Antibodies 

GR monoclonal antibody and polyclonal antibodies against PEPCK and the p65 subunit of NF-κB were purchased from Santa Cruz Biotechnology (Inc., Europe, Heidelberg, Germany). Procaspase-3 polyclonal antibodies specific and monoclonal antibodies against procaspase-9 were obtained from Cell Signaling Technology (Leiden, The Netherlands). Monoclonal antibody against β-actin was obtained from Sigma Aldrich (Sigma Aldrich, St. Louis, MO, USA). 

### 2.4. Cell Viability Assay 

MTT assay was applied, as previously described [38], to investigate the effects of essential oils on HEK-293 cell viability. Briefly, 8 × 10^3^ HEK-293 cells were plated on 96-well plates and cultured in DMEM 4.5 g/L glucose, 10% *v*/*v* FBS, 2 mM L-glutamine and 100 units/mL penicillin/streptomycin. After 24 h, cells were treated for 48 h with essential oils from oregano and *Melissa officinalis*, at concentrations of 23 μg/mL, 47 μg/mL, and 94 μg/mL, (dilution 1/40,000, 1/20,000, and 1/10,000 *v*/*v*, respectively). Essential oil of Chios Mastic was tested at concentrations of 21 μg/mL, 42 μg/mL, and 85 μg/mL (dilution 1/40,000, 1/20,000 and 1/10,000 *v*/*v*, respectively). At the same time, lavender essential oil was evaluated at concentrations of 23 μg/mL, 47 μg/mL, 94 μg/mL, and 187 μg/mL (dilution 1/40,000, 1/20,000 1/10,000, 1/5000 *v*/*v*, respectively). Control, untreated cells were incubated with DMSO at a final dilution of 1/1000 for 24 h or 48 h. Then, the medium was replaced with fresh medium containing MTT reagent at a final concentration of 0.5 mg/mL. Upon 3 h incubation and the removal of the medium, the produced formazan crystals were dissolved in 100% isopropanol, and the absorbance was measured at 570 nm and 690 nm, as a reference, in a multimode plate reader (EnSpire, PerkinElmer, Beaconsfield, UK). The intensity of the colored product (optical density, OD) is directly proportional to the number of viable cells present in the culture. Viability = Mean OD sample/Mean OD control × 100. Cell viability was expressed as the viability of the cells treated with various concentrations of the respective essential oil compared to the cell viability of the vehicle-treated (control) cells. The viability of control cells was considered 100%. 

### 2.5. Regulation of ERs, GR and NF-κB Transcriptional Activity via Essential Oils

For the evaluation of the ERs, GR and NF-κB transcriptional activities, the co-transfection of HEK-293 cells with the respective steroid receptors- or NF-κB- luciferase reporter gene constructs (currying the respective transcription factor response element at the promoter of the luciferase gene) and a β-galactosidase reporter gene construct, was performed via the application of the calcium phosphate transfection method, as previously described [39]. For assessment of ERα or ERβ transcriptional activation, HEK293 cells that expressed low levels of ERs were also co-transfected with a pEGFC2ERα or pEGFPC2ERβ construct for ERα or ERβ expression, respectively. Briefly, 3 × 10^4^ cells were grown on 24-well plates and co-transfected with either an estrogen receptor luciferase reporter gene (ER-Luc) construct and a pEGFPC2ERα or pEGFPC2ERβ construct for ERα or ERβ activity assessment, or an MMTV-GREs -Luc (Glucocorticoid Response Elements luciferase) construct for GR activity assessment, or an NF-κB-REs-Luc (NF-κB Response Elements luciferase) construct for NF-κB activity measurements and a β-galactosidase reporter construct, applying for the normalization of the results. Then, the cells were treated with the indicated amounts of essential oils in the presence or absence of (a) 10^−7^ M DEX for the GR transactivation measurements, (b) 10^−9^ M E2 for the ERs transactivation measurements, and (c) 20 ng/mL TNF-α for the evaluation of the NF-κB transcriptional activation. After 6 h incubation, cells were washed in PBSx1 and then lysed in reporter lysis buffer (Promega). The assessment of the activity of the expressed luciferase and β-galactosidase activity was followed. The light emission was measured using a chemiluminometer (LB 9508, www.berthhold.com, accessed on 5 August 2014), and the relative luciferase activity was expressed as normalized luciferase activity against β-galactosidase activity. The relative luciferase activity in control vehicle-treated cells was set as 1. Folds reduction or the induction of the luciferase activity by EOs was expressed compared to controls.

### 2.6. Western Blot Analysis 

For Western blot analysis, 2 × 10^5^ HEK-293 cells were grown on 6-well plates for 48 h in DMEM 4.5 g/L without phenol red, supplemented with 10% *v*/*v* dextran–charcoal stripped FBS. Then, cells were treated with 23 μg/mL and 47 μg/mL of the essential oils of oregano, *Melissa officinalis* and lavender, or with 21 μg/mL and 42 μg/mL of the Chios Mastic essential oil, in the presence or absence of 10 nM DEX, for 48 h. Then, cells were washed with PBSx1 and subsequently lysed in a lysis buffer containing 20 mM Tris pH: 7.5, 250 nM NaCl, 0.5% *v*/*v* Triton-X, 3 mM EDTA, supplemented with cocktail protease inhibitors. Protein determination was achieved by applying a Bradford assay, and then, cell extracts were electrophoresed on discontinuous SDS-PAGE and Western blotted, as previously described [40]. Enhanced chemiluminescence was used for the detection of protein bands. The expressed protein levels of β-actin were evaluated for the normalization of the expressed protein levels of GR, PEPCK, p65, procaspase-9 and procaspase-3. Quantification of band intensity was carried out by applying ImageJ (v1.52 p) analysis (NIH, Bethesda, MD, USA). Relative protein levels were expressed as band intensity normalized against the respective band’s intensity of β-actin. Relative protein levels in control vehicle-treated cells were set as 1.

### 2.7. Statistical Analysis 

All results are expressed as ± SD. Data were analyzed through *t*-test or two-way Analysis of Variance (two-way ANOVA), followed by Tukey’s post hoc test, using StatPlus LE 7.3.0 Software (AnalystSoft, Brandon, FL, USA). Significant differences were considered at a two-tailed *p* value < 0.05.

## 3. Results

### 3.1. Essential Oils Effects on HEK-293 Cell Viability 

**Figure 1:**
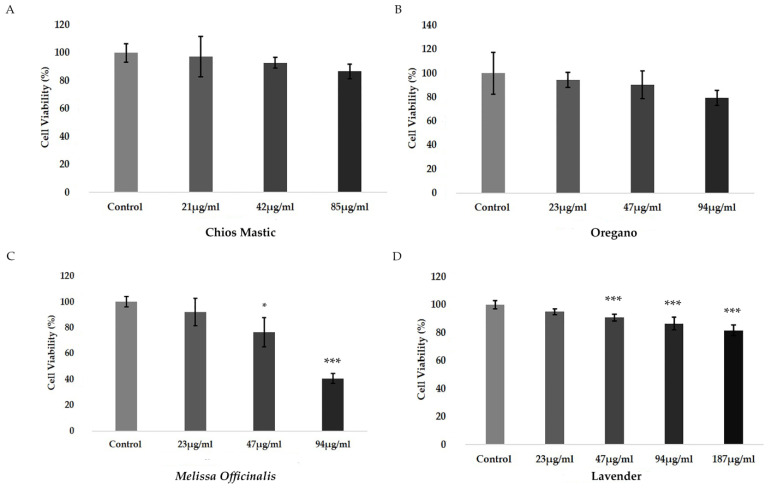
Evaluation of the cytotoxic effects of the essential oils from (**A**) Chios Mastic, (**B**) Oregano, (**C**) *Melissa officinalis* and (**D**) Lavender on HEK-293 cells. Cytotoxicity was assessed by applying MTT assay to HEK-293 cells subjected to essential oils treatment for 48 h. Viability of control vehicle-treated cells was considered 100%. Relative cell viability (Cell viability, %) is expressed as the viability of the cells treated with various concentrations of the respective essential oil compared to the viability of the control cells. Data were analyzed by *t*-test and are expressed as mean ± SD (n = 5), * *p* < 0.05, *** *p* < 0.001 compared to the respective control.

To evaluate the possible effect of the essential oils on HEK-293 cells viability, an MTT assay was applied. HEK-293 cells were incubated with essential oils at a concentration range of 23 μg/mL to 187 μg/mL for 48 h, as indicated in Figure 1. As shown in Figure 1A,B, no statistically significant reduction in cell viability was observed in the Chios Mastic essential oil and the oregano essential oil at a concentration range of 21 to 85 μg/mL (Figure 1A) and 23 to 94 μg/mL (Figure 1B), respectively. Lavender essential oil (Figure 1D) showed moderate reduction in cell viability by up to approximately 10–20%, at a concentration range from 47 μg/mL to 187 μg/mL (Figure 1D), whereas the highest cytotoxicity was observed in the *Melissa officinalis* essential oil (Figure 1C), which exhibited 35% and 50% reduction in cell viability at concentration of 47 μg/mL and 94 μg/mL, respectively, compared to the control vehicle (1/1000 *v*/*v* DMSO) treated cells.

### 3.2. Chios Mastic Essential Oil Caused Moderate Reduction in the E2-Induced Transcriptional Activation of ERα 

**Figure 2:**
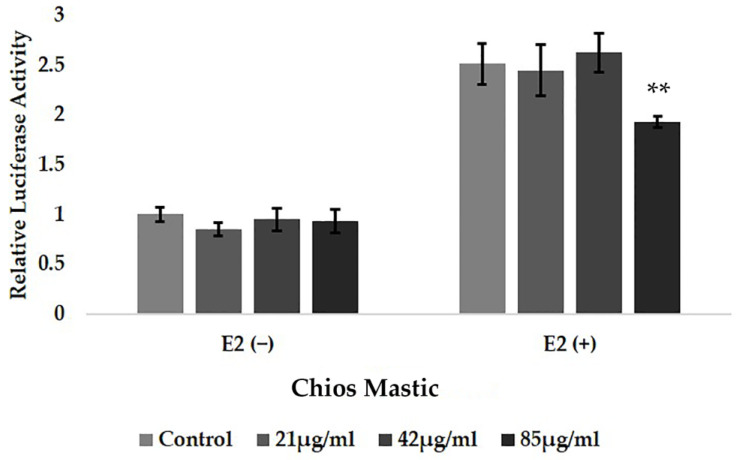
Chios Mastic essential oil caused moderate reduction in the E2-induced ERα transcriptional activation. Cells grown in hormone-free medium for 48 h were transiently co-transfected with an ERE-dependent Luciferase reporter gene construct, a PEGFPC2ERα and a β-galactosidase reporter construct. Subsequently, cells were treated with the indicated amounts of the Chios Mastic essential oil, in the presence or absence of E2, for 6 h. Then, cells were harvested and lysed. Assessment of the luciferase and β-galactosidase activity was followed in cell extracts. Relative luciferase activity was expressed as normalized against the β-galactosidase activity. Relative luciferase activity in control cells was set as 1. Data were analyzed by two-way ANOVA and are expressed as mean ± SD, (*n* = 6), ** *p* < 0.01.

Taking into account the crucial role of steroid hormones, especially that of the glucocorticoids and estrogens, in the regulation of immune responses in conjunction with the emerging anti-inflammatory activities of essential oils, especially that of the mastic essential oil [41], we studied the possible interference of the mastic essential oil with the regulation of the ERs transcriptional activation. Thus, ERα transcriptional activation was evaluated in the presence or absence of the Mastic essential oil at a concentration range of 21 to 85 μg/mL and/or E2 10^−9^ M, applying ERE-dependent luciferase reporter gene assay, as described in the experimental section. As shown in Figure 2, Chios Mastic essential did not induce ERα transcriptional activation. A negligible suppression, by up to 20%, of the E2-induced ERα transcriptional activation was observed in Mastic essential oil at the highest concentration examined (85 μg/mL). Similarly, no statistically significant effect on the ERβ transcriptional activation was observed in the Mastic essential oil. Likewise, no effect on the E2-induced ERα transcriptional activation was observed in the essential oils from lavender, oregano and *Melissa officinalis* (Supplementary Figure S1). Thus, the involvement of the essential oils’ effect on ERs signaling was not further evaluated. 

### 3.3. Chios Mastic, Oregano, Melissa officinalis and Lavender Essential Oils Regulate the DEX-Induced Transcriptional Activation of GR

**Figure 3:**
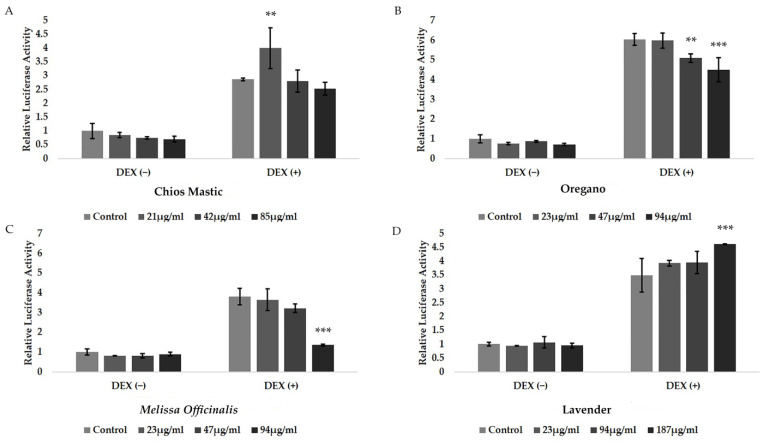
Regulation of the DEX-induced GR transcriptional activation by the essential oils from (**A**) Chios Mastic, (**B**) Oregano, (**C**) *Melissa officinalis*, and (**D**) Lavender in HEK-293 cells. Cells grown in hormone-free medium were transiently co-transfected with a GRE-Luc reporter gene construct and a β-galactosidase reporter construct. Subsequently, cells were treated with the indicated amounts of the essential oils, in the presence or absence of 1 μΜ DEX, for 6 h. Then, cells were harvested and lysed. Assessment of the luciferase and β-galactosidase activity in cell extracts was followed. Relative luciferase activity was expressed as normalized against β-galactosidase activity. Relative luciferase activity in control cells was set as 1. Data were analyzed by two-way ANOVA and are expressed as mean ± SD, (*n* = 6), ** *p* < 0.01; *** *p* < 0.001.

Since we did not observe a significant effect of essential oils on ER activity, the possible effect of the essential oils on the transcriptional regulation of GR was assessed by applying a GRE-dependent luciferase reporter gene and β-galactosidase reporter gene assay to the HEK-293 cells. Thus, HEK-293 cells grown in hormone-depleted medium for 48 h were subsequently co-transfected with the respective constructs, as described in the experimental section. Then, cells were subjected to treatment with the essential oils at no cytotoxic concentrations, as indicated by the cytotoxicity assessment upon 24 h treatment (Supplementary Figure S2), in the presence or absence of 1 μΜ DEX for 6 h (Figure 3). As shown in Figure 3, DEX induced 3.5–5 fold increases in GR transcriptional activity, as was expected, while neither the induction nor suppression of GR transcriptional activation was observed in terms of the essential oils. Interestingly, oregano (Figure 3B) and *Melissa officinalis* (Figure 3C) essential oils suppressed the DEX-induced GR transcriptional activation. Specifically, the oregano essential oil caused approximately 15% and 25% suppression of the DEX-induced GR transcriptional activation at the concentrations of 47 μg/mL and 94 μg/mL, respectively. Essential oil from *Melissa officinalis* exhibited the highest suppressive effect, causing a 60% reduction in the DEX-induced GR transcriptional activation at a concentration of 94 μg/mL. On the contrary, a synergistic effect on the DEX-induced GR transcriptional activation was observed in the essential oil from lavender, leading to a statistically significant increase in DEX-induced GR transcriptional activation, by 25%, at a concentration of 184 μg/mL (Figure 3D). A similar effect (increase by 30%) was also observed in the Chios Mastic essential oil at the low concentration of 21 μg/mL (Figure 3A).

### 3.4. Effect of Essential Oils from Chios Mastic, Oregano, Melissa officinalis and Lavender on GR and PEPCK Protein Levels

**Figure 4:**
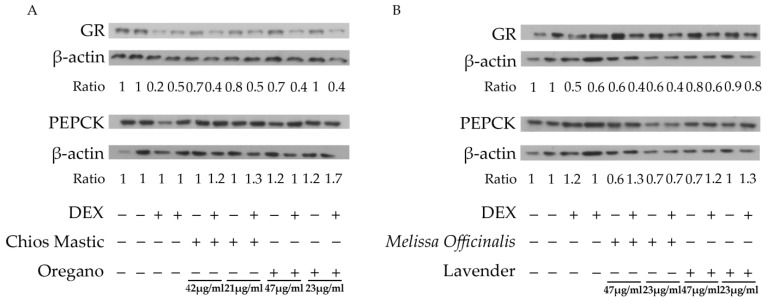
Regulation of GR and PEPCK protein levels by the essential oils from (**A**) Chios Mastic and Oregano, (**B**) *Melissa officinalis* and Lavender. Cells grown in hormone-free medium were incubated with the essential oils in the absence or presence of DEX 10^−8^ M, for 48 h in a hormone-free medium. Data were expressed as the ratios of bands intensity of GR and PEPCK normalized against the respective band’s intensity of β-actin. Relative band intensity of control cells was set as 1. The uncropped blots are shown in Figure S4.

To further analyze the potential effects of the essential oils on GR signaling and to investigate the possible dissociative activity of the essential oils on GR transactivation (and thus GR gluconeogenic activities) and transrepression (suppression of the TNF-α- induced NF-κB activation, and thus GR anti-inflammatory activities), comparative studies of essentials oils effect on the regulation of GR and PEPCK protein levels was performed, applying Western blot analysis. Interestingly, as it is shown in Figure 4, essential oils induced a decrease in GR protein levels. More specifically, Chios Mastic and oregano essential oils caused a 30% reduction in GR protein levels at a concentration of approximately 45 μg/mL. A reduction in GR protein levels by 20% was also observed in the Chios Mastic oil at the lower concentration of 21 μg/mL (Figure 4A). Similarly, essential oil from *Melissa officinalis* caused a 40% reduction in GR protein levels at the concentration range from 23 μg/mL to 47 μg/mL. Reduction in GR protein levels by the oregano essential oil and the *Melissa officinalis* essential oil may be associated with the respective essential oils’ observed suppression of the DEX-induced GR transcriptional activation. Moreover, a 20% reduction in GR protein levels was also induced by the lavender essential oil at a concentration of 47 μg/mL (Figure 4B). A further decrease, by 30%, in GR protein levels was observed in the presence of DEX. 

In the same context, the potential role of essential oils in the regulation of PEPCK, which constitutes a GR target and a key gluconeogenic enzyme [42,43], was assessed. In accordance with the essential oils-induced reduction in GR protein levels, *Melissa officinalis* and lavender essential oils caused a reduction in PEPCK protein levels. Specifically, *Melissa officinalis* essential oil caused 30% and 40% reductions in PEPCK protein levels at the concentrations of 23 μg/mL and 47 μg/mL, respectively (Figure 4B). Lavender essential oil caused a 30% reduction in PEPCK protein levels at the concentration of 47 μg/mL (Figure 4B). Co-administration with DEX reversed the suppressive effect of *Melissa officinalis* essential oil on the PEPCK protein level, causing a 30% increase compared to the control, (70% increase compared to the *Melissa officinalis*-induced reduction) at a concentration of 47 μg/mL. A similar effect was observed during the co-administration of lavender essential oil with DEX. Thus, an increase in PEPCK protein levels was observed to be approximately 20–30% at a concentration range of 23–47 μg/mL of lavender essential oil (Figure 4B) when administered with DEX compared to controls (60% increase compared to the lavender-induced reduction). A similar effect on the induction of PEPCK protein expression was observed upon co-administration of Chios Mastic essential oil with DEX (Figure 4A), supporting the observed strengthening effect of the lavender (Figure 4B) and Chios Mastic (Figure 4A) essential oils on the DEX-induced GR transcriptional activation. At both concentrations examined, essential oil from Chios Mastic did not cause any effect on PEPCK protein levels, whereas essential oil from oregano caused a 20% increase compared to controls (Figure 4A). 

### 3.5. Anti-Inflammatory Activities of Essential Oils from Chios Mastic, Oregano and Melissa officinalis via Suppression of NF-κB Transcriptional Activation

**Figure 5:**
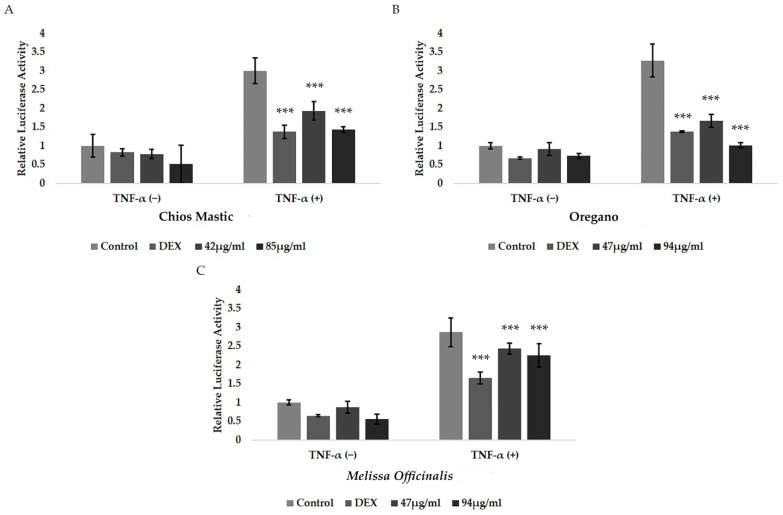
Anti-inflammatory activities of the essential oils from the Chios Mastic, Oregano and *Melissa officinalis*. Suppression of the TNF-α induced NF-κB transcriptional activation by the essential oils from the (**A**) Chios Mastic, (**B**) Oregano and (**C**) *Melissa officinalis*. HEK-293 cells were co-transfected with the NF-κB-luciferase reporter and the β-galactosidase reporter constructs and subsequently were treated with the indicated concentrations of the essential oils, or with 1 μM DEX, for 6 h, in the presence or absence of 20 ng/mL TNF-α, at hormone-free medium. Assessment of the luciferase and the β-galactosidase activity was followed. Results were expressed as relative luciferase activity normalized against β-galactosidase activity. Relative luciferase activity in control cells was set as 1. Data were analyzed by two-way ANOVA and are expressed as mean ± SD, (*n* = 6), *** *p* < 0.001.

To evaluate the possible anti-inflammatory activities of the essential oils from Chios Mastic, oregano and *Melissa officinalis*, an NF-κB-associated luciferase/β-galactosidase reporter gene assay was applied. Thus, the effect of the essential oils on the TNF-α-induced NF-κΒ transcriptional activation was assessed. Results from the study revealed the suppressive effect of the essential oils on the TNF-α-induced NF-κΒ transcriptional activation, uncovering their potential anti-inflammatory activities. Specifically, essential oil from Chios Mastic caused approximately 40% and 60% statistically significant inhibition of the TNF-α-induced NF-κΒ transcriptional activation at the concentrations of 42 μg/mL and 85 μg/mL, respectively (Figure 5A). Similarly, statistically significant suppression of the TNF-α-induced NF-κB transcriptional activation, by approximately 50% and 70% (Figure 5B), by oregano essential oil at concentrations of 47 μg/mL and 94 μg/mL, respectively, was observed. Essential oil from *Melissa officinalis* also exhibited suppression of the TNF-α-induced NF-κΒ transcriptional activation, although to a lower extent. Thus, essential oil from *Melissa officinalis* caused approximately 15% and 30% reduction in the NF-κB transcriptional activation at concentrations of 47 μg/mL and 94 μg/mL, respectively (Figure 5C). No anti-inflammatory activity was observed in the Lavender essential oil at a concentration range of 23 to 94 μg/mL (Supplementary Figure S3). 

**Figure 6:**
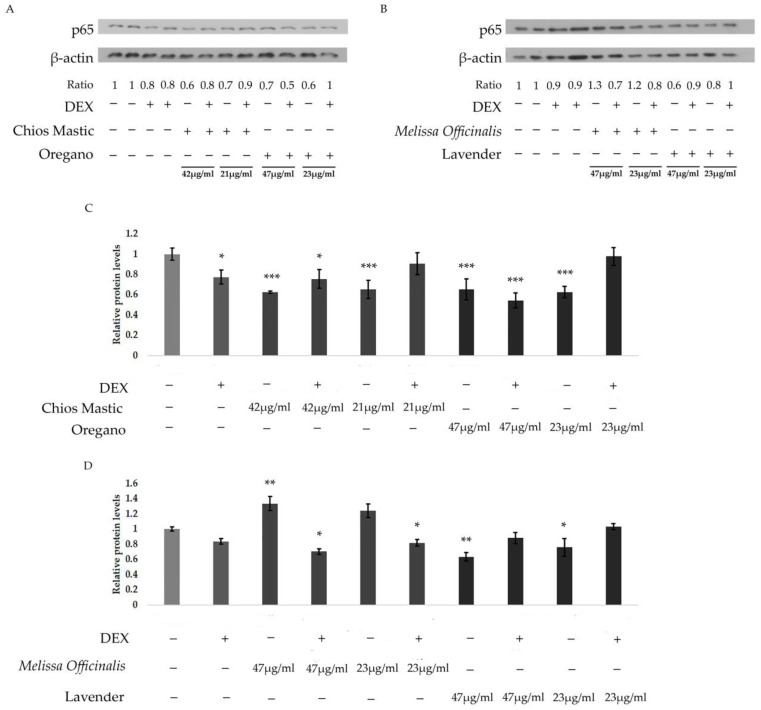
Regulation of the p65 protein levels by essential oils. Representative images from Western blot analysis of the p65 subunit of NF-κB in HEK-293 cells treated for 48 h in a hormone-free medium with the essential oils from (**A**) Chios Mastic, at concentrations of 21 μg/mL and 42 μg/mL, and with the essential oil from oregano, and (**B**) Melissa officinalis and lavender, at concentrations of 23 μg/mL and 47 μg/mL. Results were expressed as the ratios of the p65 bands intensity normalized against the respective band intensity of the β-actin. Relative band intensity of control cells was set as 1. (**C**,**D**) Quantification of the results in A and B, respectively. Data are expressed as means of the ratios ± SD, (*n* = 3), * *p* < 0.05; ** *p* < 0.01; *** *p* < 0.001, compared to control vehicle-treated cells. The uncropped blots are shown in Figure S4.

To evaluate whether the EOs-induced suppression of the NF-κB transcriptional activity was associated with the regulation of the protein levels of the p65 subunit of NF-κΒ, Western blot analysis of p65 was performed in protein extracts from HEK-293 cells treated with essential oils at a concentration range indicated in Figure 6, in the absence or presence of DEX for 48 h. As shown in Figure 6, a reduction in p65 protein levels was observed in the essential oils from Mastiha resin, oregano and lavender by 20–40% at a concentration range of approximately 22 to 45 μg/mL. Essential oil from *Melissa officinalis* showed an increase in p65 protein levels, which is in accordance with its just moderate activity on the suppression of the NF-κB activity. 

### 3.6. Regulation of the Mitochondrial-Dependent Apoptosis by the Essential Oils 

**Figure 7:**
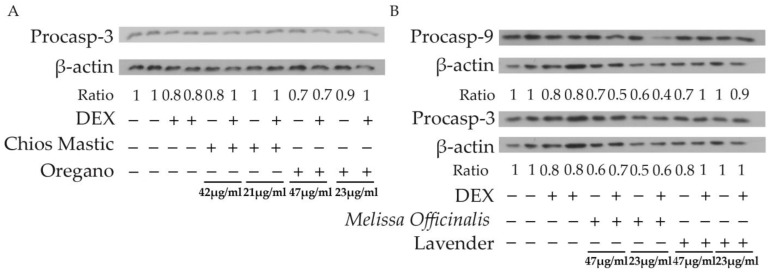
Evaluation of pro-apoptotic actions of the essential oils in HEK-293 cells. Western blot analysis of procaspase-9 and procaspase-3 in extracts from HEK-293 cells treated with essential oils ((**A**), Chios Mastic and Oregano; (**B**), *Melissa Officinalis* and Lavender), at the indicated concentrations, in the absence or presence of DEX for 48 h. Data are expressed as ratios of the levels of the apoptosis-associated molecules against the respective levels of β-actin. Relative band intensity of control vehicle-treated cells was set as 1. The uncropped blots are shown in Figure S4.

Furthermore, to assess the possible pro-apoptotic activities of the essential oils, Western blot analysis of procaspase-3 and procaspase-9 protein levels was performed in extracts from HEK-293 cells treated with essential oils, at the indicated concentrations (Figure 7), and/or 10 nM DEX for 48 h, in hormone-free medium. As shown in Figure 7B, 47 μg/mL of essential oils from lavender and *Melissa officinalis* showed a 30% reduction in procaspase-9 protein levels. This effect was also accompanied by a reduction in procaspase-3 protein levels. More specifically, 47 μg/mL of essential oil from lavender caused a 20% reduction in procaspase-3 protein compared to the control. *Melissa officinalis* essential oil also exhibited 20% to 50% reductions in procaspase-3 protein levels. Essential oils from Chios Mastic and oregano showed no remarkable effects on the procaspase-9 protein levels. In contrast, 42 and 47 μg/mL of the essential oils of Chios Mastic and Oregano caused 20% and 30% reduction in caspase-3 levels, respectively (Figure 7A). 

## 4. Discussion

In recent years, scientific studies that demonstrate the therapeutic benefits of chemical compounds of plant origin and particularly of essential oils from plants have been constantly growing [1,32,44,45,46,47]. In this study, four Greek essential oils were assessed for their biological actions in the HEK-293 cell line. Specifically, Chios Mastic, *Melissa officinalis*, oregano and lavender essential oils have been investigated with respect to their anti-proliferative and anti-inflammatory actions and their possible interference with estrogen and glucocorticoid receptors signaling. GRs and ERs are crucial regulators of cell physiology, affecting many cellular functions, including growth and development, cellular metabolism, and apoptosis [14,48,49]. In addition, those receptors are well-known for their anti-inflammatory actions via interference with NF-κB signaling and the control of the expression of many inflammation-related genes. Moreover, glucose homeostasis and lipid and protein metabolism are highly affected by glucocorticoids and estrogens [50,51]. Considering the high use of glucocorticoids for pharmaceutical purposes, due to their strong anti-inflammatory actions, which are accompanied by increased glucose synthesis, the possible interference of essential oils with the steroid hormone receptors signaling may uncover novel plant-derived steroids-like compounds as lead molecules for the development of selective steroid receptor regulators with increased desired anti-inflammatory activities but with reduced adverse side effects, such as increased glucose synthesis, myopathy, osteoporosis and hypothalamus–pituitary–adrenal axis dysregulation [10,52]. 

In this frame, comparative studies on the anti-inflammatory activities and interference with the glucocorticoid signaling of essential oils from Chios Mastic, *Melissa officinalis*, oregano and lavender were performed. 

Applying luciferase assay and Western blot analysis, the effect of essential oils on the TNF-α-induced NF-κΒ activation and the regulation of the protein levels of the p65 subunit of NF-κΒ were evaluated. Our results showed a suppressive effect on the TNF-α-induced NF-κΒ activation by the essential oils from Chios mastic, oregano and *Melissa officinalis*. Specifically, essential oils from Chios mastic and oregano were the most active ones, whereas essential oil from *Melissa officinalis* exhibited lower activity. The anti-inflammatory activity of the essential oils could be attributed to essential oils compounds, such as α-thujone, β-thujone, camphor, caryophyllene and terpenoids that have been reported to exert suppression of NF-κB activity [53,54]. Interestingly, essential oils from oregano and Chios mastic, which were the most active ones, are also the most enriched in thujene and camphene, respectively (Supplementary Data S1, Essential oils chemical composition). Moreover, the reduction in NF-κB activity by the Chios mastic and oregano essential oils was accompanied by a reduction in the p65 subunit of NF-κB. Thus, the suppressive effect of the essential oils from Chios mastic and oregano on the NF-κB activity could be attributed to essential oils’ suppressive effect both on NF-κΒ transcriptional activation and protein levels. In the same frame, *Melissa officinalis*, which showed limited anti-inflammatory action, caused an increase in p65 protein levels. 

As regards the interference of essential oils with glucocorticoid signaling, and thus the regulation of NF-κΒ signaling and glucose synthesis, our results showed that essential oils did not induce GR transactivation. For the first time, our study revealed that essential oils from Chios Mastic and Lavender caused a moderate increase in DEX-induced GR transcriptional activation. However, essential oils from oregano and *Melissa officinalis* suppressed the DEX-induced GR transcriptional activation, in accordance with previous observation [55,56]. The enhancement of DEX-induced GR transcriptional activation by Chios mastic oil may lead to the glucocorticoid-induced regulation of inflammatory and pro-inflammatory molecules expression, which is also responsible for the anti-inflammatory actions of GR [57]. Moreover, for the first time, we showed a reduction in GR protein levels by the essential oils. Considering that GR is involved in the regulation of NF-κB activity, reduction in GR protein levels may also affect essential oils’ anti-inflammatory actions. As regards the interference of essential oils with glucocorticoids and thus glucose synthesis, our results revealed the regulation of GR and its target PEPCK protein levels by the essential oils. Particularly, a reduction in GR protein levels was observed in all the essential oils examined, indicating that this action may constitute a common action of essential oils. Chios mastic oil’s suppressive effect on GR protein levels may compensate for its promoting effect on GR transcriptional activation, resulting in no effect of Chios mastic essential oil on PEPCK protein levels, and thus no induction of glucose synthesis. A decrease in GR protein levels by essential oils from oregano, *Melissa officinalis*, and lavender was followed by a decrease in PEPCK protein levels, highlighting essential oils’ potential anti-gluconeogenic actions. An antagonistic effect of the synthetic glucocorticoid dexamethasone on this action was uncovered, as regards *Melissa officinalis* and lavender essential oil. Thus, the reversal of the essential oils-induced decrease in PEPCK was observed when administered with DEX, corroborating the possible interference of the essential oils with glucocorticoids signaling. A reduction in GR protein levels by the oregano and *Melissa officinalis* essential oils may be responsible for the suppressive effect of the oils on the DEX-induced GR transcriptional activation. The interference of essential oils with glucocorticoids signaling is also in accordance with data from the literature demonstrating that essential oils’ compounds such as α-Pinene, limonene, α-thujene, myrcene, sabinene, and para-cymene, are responsible for the calming effect of essentials oils via the interference and suppression of the HPA axis [53,58,59]. In this context, lavender essential oil is shown to cause a reduction in the stress hormone cortisol [60,61,62]. Thus, the effect of essential oils on GR signaling could be exerted both via the regulation of glucocorticoid levels and GR transcriptional activation and/or protein levels. A similar antagonistic action of essential oils compounds on steroid signaling has been observed using germacrene analogs, which have been proposed to exert anti-androgenic activities [63]. Lavender essential oil is also proposed to be involved in prepubertal gynecomastia via its anti-androgenic and estrogenic activities [64,65]. The interference of lavender essential oil with estrogen signaling is proposed to be beneficial for perimenopausal women by inducing an increase in estrogen levels and relieving perimenopausal symptoms [66]. Docking analysis verified the ability of thymol and carvacrol, compounds of essential oils, to bind to estrogen receptors [67]. Nevertheless, the estrogenic activity of lavender was not confirmed in a rat model [68] and in hormone-dependent (MCF-7) and -independent (MDA-MB-231) cell lines [69]. In the same frame, in this study, no interference of Chios Mastic, *Melissa officinalis*, oregano and lavender essential oils with estrogen signaling, either by ERα or ERβ, was found.

Essential oils are also well-known for their anti-proliferative and apoptotic activities. In this study, the MTT assay revealed cytotoxic activities of essential oils from *Melissa officinalis* and lavender, whereas essential oils from Chios Mastic and oregano showed no cytotoxicity at a concentration range from approximately 20 μg/mL to 85 μg/mL. Similarly, caspase-3 activation was not observed in the Chios Mastic and oregano essential oil. However, in accordance with the evaluation of the essential oils’ cytotoxic effects, caspase-3 activation was observed in HEK-293 cells treated with *Melissa officinalis* and lavender at a concentration range from 47 to 187 μg/mL, indicating apoptosis activation. Most importantly, caspase-3 activation was accompanied by caspase-9 activation, revealing mitochondrial-dependent apoptosis activation. The lavender essential oil-induced caspase-9 activation supports previously reported observations applying annexin staining [31]. Chios Mastic and oregano essential oils also induced caspase-9 activation. Glucocorticoids are well known to induce mitochondrial-dependent apoptosis in a tissue-specific manner, including epithelial cells of the digestive system [16]. Thus, the apoptotic activities of the essential oils from lavender, Chios Mastic and oregano might be exerted, among others, via interference with glucocorticoid signaling and could have application in glucocorticoid-based cancer treatment. Anti-proliferative activities of essential oils from oregano are also reported in the literature [34,70,71] and may be associated with their effect on the induction of apoptosis via the intrinsic and/or extrinsic pathway. Moreover, there is supporting evidence for essential oils’ compounds like carvacrol, limonene, citral, thymol and terpenoid analogues, such as Terpinen-4-olto are involved in the apoptotic mechanism in many cell types. Characteristic examples are murine mesothelioma (AE17), melanoma cells (B16-F10), and fibroblasts (L929), colon cancer (LS174T) cells, breast cancer (MCF-7) cell line, human metastatic breast cancer (MDA-MB 231) cell line and human promyelocytic leukemia (HL-60) cells [70,72]. 

To conclude, in this comparative study, the anti-inflammatory and apoptotic activities of the Greek Oregano, *Melissa officinalis*, Lavender and Chios Mastic essential oils were evaluated in relation to steroid hormones signaling interference. Estrogenic activity of the essential oils was not detected, whereas interference with glucocorticoid signaling was observed, affecting both glucocorticoid receptor activity and protein levels. This action also came with the regulation of the GR target gene expression, uncovering potential anti-gluconeogenic activities of the essential oils via possible interference with glucocorticoid signaling. The suppression of TNF-α-induced NF-κΒ activation that was not accompanied by the activation of GR transcriptional activation may possibly indicate essential oils’ potential applications to the treatment of immune system disorders, minimizing the adverse side effects of glucocorticoids. Moreover, the anti-inflammatory activities of essential oils are revealed to be exerted both by suppression of the NF-κB activity and NF-κB protein levels. Similar to glucocorticoids, the apoptotic activities of the essential oils are exerted, at least in part, via activation of the mitochondrial-dependent apoptosis. Interestingly, a comparative evaluation of the biological actions of EOs revealed that Chios Mastic (Mastiha) and oregano EOs exhibited considerable anti-inflammatory activities. The former showed a reduction both in NF-κB activity and protein levels. Mastic oil also caused a reduction in GR protein levels that may compensate for its boosting effect on the DEX-induced GR transcriptional activation, ending up in no induction of the gluconeogenic PEPCK protein levels that constitute a GR target. Oregano, *Melissa officinalis* and lavender EOs suppressed the transcriptional activation of the GR. Furthermore, the most active, *Melissa officinalis* EO, showed a reduction both in GR and PEPCK protein levels. Thus, the anti-inflammatory and anti-gluconeogenic activities of the EOs were uncovered, possibly via the regulation of GR signaling. Moreover, the cytotoxic actions of *Melissa officinalis* and lavender essential oils via the induction of mitochondrial-dependent apoptosis were revealed. Our results highlight these essentials oils’ anti-inflammatory, anti-gluconeogenic and apoptotic actions in relation to their implication on the regulation of steroid hormones actions, uncovering their use in steroid therapy with many potential applications in pharmaceutical and health industries as anti-cancer, anti-hyperglycemic and anti-inflammatory supplements.

## Data Availability

All data, tables, and figures are original. Details on data analysis are available from the corresponding author upon reasonable request.

## Supplementary Materials

The following supporting information can be downloaded at: https://www.mdpi.com/article/10.3390/life13071534/s1, Supplementary Data S1: Chemical composition of essential oils; Supplementary Figure S1: Effect of essential oils on ERs transcriptional activation; Figure S2: Cytotoxic effects of essentials oils upon 24 h incubation; Figure S3: Effect of lavender essential oil on NF-κB transcriptional activation. Figure S4: Representative images used in western blot analysis.

## Abbreviations

ATCC	American type culture collection
DEX	Dexamethasone
DMEM	Dulbecco’s Modified Eagle Medium
DMSO	Dimethyl Sulfoxide
E2	Estradiol
EOs	Essential Oils
ER	Estrogen receptor
ERE	Estrogen Response Elements
ERα	Estrogen receptor alpha
ERβ	Estrogen receptor beta
FBS	Fetal Bovine Serum
G6Pase	Glucose 6-phosphatase
GR	Glucocorticoids Receptor
GREs -Luc	Glucocorticoid Response Elements-luciferase
HEK-293	Human Embryonic Kidney-293
HIF-1α	Hypoxia inducible factor 1 alpha
HPA	Hypothalamus-Pituitary-Adrenal
IL-6	Interleukin-6
NF-κB	Nuclear Factor Kappa B
NF-κB-REs-Luc	NF-κB Response Elements-luciferase
OD	Optical Density
PEPCK	Phoshoenolpyruvate carboxykinase
ROS	Reactive Oxygen Species
TNF-α	Tumor Necrosis factor alpha
