# Supplementary material for: Comparative Studies on the Anti-Inflammatory and Apoptotic Activities of Four Greek Essential Oils: Involvement in the Regulation of NF-κΒ and Steroid Receptor Signaling
Source: Life (Basel). 2023 Jul 10;13(7):1534. doi: 10.3390/life13071534 (PMC10381560; doi:10.3390/life13071534)
Supplement: Supplementary file 1 [file life-13-01534-s001.zip › Supplementary Data S1. Chemical composition of essential oils.pdf]

ΕΝΩΣΗ
ΜΑΣΤΙΧΟΠΑΡΑΓΩΓΩΝ ΧΙΟΥ
THE CHIOS GUM MASTIC
GROWERS ASSOCIATION

Chios Mastic Oil – Chios Mastiha Oil.

Technical Specifications

Number: SP551
Edition: 8
Date of issue: 12/10/2018

---

1. Product Identification

Product Code:	*TMX-0001*
Product Name:	*Chios* Mastic Oil (Chios Mastiha Oil)
Natural product:	*Yes*

---

2. Product Description

Chios Mastic Oil comes out through distillation process with water, it is **100% natural**, does not contain any harmful substance or any additives, which could have damaging effects for the consumer.

***Natural occurrence*:**	From Chios Mastic Gum (Chios Mastiha) the air-dried resinous exudation from *Pistacia. Lentiscus* L. var. Chia (Family Anacardiaceae), a shrub of small evergreen tree. The tree is native to the Mediterranean region and principally cultivated on the Greek island of Chios.
CAS Name:	Lentisque absolute
CAS Number:	61789-92-2
EINECS:	263-098-6
**INCI NAME:**	MonoID: 24054 Pistacia Lentiscus (Mastic) Gum Oil

---

3. Food Legislation Information

The Council of Europe (1981) included mastic in the list of plants and parts thereof, which are acceptable for use in foods (annex I, EC 2081/1992 article 1, part1).

Chios Mastic Oil is manufactured according to the legislation at present in force, in this country and the specifications of this company, being fit for human consumption at the

Κ. ΜΟΝΟΜΑΧΟΥ 1
Τ.Θ. 110, ΧΙΟΣ 82100
ΤΗΛ.: 22710 21001-2-3
FAX: 22710 26700
e-mail:exports@gummastic.gr
www.gummastic.gr

1, K. MONOMACHOU str
P.O. BOX 110
82100 CHIOS, GREECE
TEL.: +30 22710 21001-2-3
FAX: +30 22710 26700
VAT: EL 096000437

dose recommended for its use. The usage of this product in certain applications depends on local interpretations concerning foodstuffs.

---

4. Product Characteristics

Organoleptic Properties:

Physical form:	Resinous liquid.
Colour:	Colourless to faintly yellow.
Odour:	Balsamic, green and rustic odour.

Physical Properties:

Density $\{d_{20}\}$:	0,8300 - 0,8700 gr/ml (20 °C)
Flash point:	+105 °C

(Parameters not routinely checked)

Reactive Index $\{n_D^{20}\}$:	1,4640 - 1,4680
Optical Rotation $[\alpha]_D$:	+35 ° - +40°
Chromatic Parameters:	L: 90.00 - 105.00
	a: -15.00 - 5.00
	b: 0.00 - 25.00
Total Acidity (as oleic acid):	0,06 – 0,09 %
Saponification Value:	*
Freezing Point:	below 0 °C

Typical Chemical Composition*:

Evaluated by gas chromatography – with flame ionization detector (GC-FID) technique

Compound	Composition (%)
a-pinene	79-88
β-myrcene	7-14
β-pinene	2,0-3,0
Limonene	0,40-0,95
β-caryophyllene	0,15-0,65
Camphene	0,30-0,8

* Compounds with concentration above 0,5% referred only. The aforementioned concentration limits is based on data available to us, however they should not be account as strictly quality specifications, since Mastic Oil is natural product, very sensible in quality and weather condition variations, which may affect significantly the concentration of its main ingredients.

Κ. ΜΟΝΟΜΑΧΟΥ 1
Τ.Θ. 110, ΧΙΟΣ 82100
ΤΗΛ.: 22710 21001-2-3
FAX: 22710 26700
e-mail:exports@gummastic.gr
www.gummastic.gr

1, K. MONOMACHOU str
P.O. BOX 110
82100 CHIOS, GREECE
TEL.: +30 22710 21001-2-3
FAX: +30 22710 26700
VAT: EL 096000437

Microbiological Properties:

Chios Mastic Oil does not contain any pathogenic organism or their metabolic products in amounts, which is harmful to the health of the final consumer. Additionally, this product presents antimicrobial and antifungal activity independently confirmed and published in scientific papers.

Parameter	Method of Analysis	Result
Aerobic Microbial Count at 30°C, cfu/g	APHA 6.54	<1000
Total coliforms, cfu/g	APHA 6.54	< 100
Escherichia Coli, cfu/g	APHA 6.54	<10
Yeast-Molds, cfu/g	APHA 6.54	<10

Heavy Metals:

Parameter	Method of Analysis	Result
Cadmium, mg/kg	ICP-OES	<0.01 mg/Kg
Lead, mg/kg	ICP-OES	<0.05 mg/Kg
Mercury, mg/kg	MTD Me.31	<0.02 mg/Kg
Arsenic, mg/kg	MTD Me.41	<0.05 mg/Kg

5. Danger identification

No specific hazard known.

6. Shelf Life

Stable for 24 month if stored tightly closed in the original containers, in a dry & cool place (< 7°C), away from light.

7. Safety & Handling Instructions

Handle in well ventilated areas, avoid eyes or skin contact. Keep it away from source of heat, sparks and naked flame. It is advised to use glasses and gloves, not to breathe the emitted vapours. Close container tightly after using.

8. Packaging:

Chios Mastic Oil is supplied in glass jars or aluminium internal lacquered containers.
Net Weights: 5, 10, 50, 100, 500, 1000g

9. Additional Data:

- Chios Mastic Oil exhibits a high level of fluidity.
- Chios Mastic oil presents antimicrobial and antifungal activity.
- The product does not contain (with reference to EC 1829/2003& 1830/2003) Genetically Modified Organisms and/or any products derived from such organisms.

This product corresponds with the regulations of the European food legislations. This specification is carefully drawn up and represents our current knowledge. However, we are not liable for any damage resulting from wilful acts or gross negligence. We guarantee therefore a proper quality within our general conditions of sales.

HELLENIC AGRICULTURAL ORGANIZATION - DEMETER
DIRECTORATE GENERAL OF AGRICULTURAL RESEARCH N.AG.RE.F.

INSTITUTE OF PLANT BREEDING AND GENETIC RESOURCES
DEPARTMENT OF AROMATIC AND MEDICINAL PLANTS
P.O.Box 60458, GR 57001, THERMI, THESSALONIKI, GREECE
TEL.: +30 2310 471 110, ext. 0 202
FAX: +30 2310 473 024
Email: chatzopoulou@ipgrb.gr
aromparc.ethiage@gmail.com

Thessaloniki, 24 July 2019
Prot.no: 2944

To THARROS SA – TZIOUMAKIS Bros
5th km Tirnavos-Larisa
GR 40100, TIRNAVOS
GREECE

CERTIFICATE OF ANALYSIS

Sample number	:	186/2019
Sample type	:	LAVENDER ESSENTIAL OIL
Costumer	:	THARROS SA – TZIOUMAKIS Bros
Region	:	TIRNAVOS – LARISA, GREECE

ESSENTIAL OIL ANALYSIS

COMPOUND	% YIELD	COMPOUND	% YIELD
hexyl methyl ether	0,08	hexyl propionate	0,07
α-thujene	0,15	1-octen-3 yl acetate	1,14
α-pinene	0,31	3-octanyl acetate	0,04
camphene	0,12	allo-ocimene	0,22
1-octen-3-ol	0,15	camphor	0,16
3-octanone	0,49	hexyl isobutyrate	0,10
β-myrcene	1,68	lavandulol	0,58
3-octanol+butyl butyrate	0,14	borneol	0,42
alpha -phellandrene	0,07	terpinen-4-ol	6,76
δ-3-carene	0,10	crypton	0,08
n-hexyl acetate	0,53	α-terpineol+hexyl butyrate	1,50
α-terpinene	0,12	linalyl acetate	26,92
p-cymene	0,12	bornyl acetate	0,15
limonene	0,46	lavandulyl acetate	5,28
β-phellandrene	0,07	neryl acetate	0,57
1,8-cineol	0,61	geranyl acetate	1,27
cis-β-ocimene	9,72	β-caryophyllene	4,34
trans-β-ocimene	3,93	α-santalene	0,33
γ-terpinene	0,29	α-bergamotene	0,14
cis-linalool oxide	0,16	α-humulene	0,12
α-terpinolene	0,17	trans-β-farnesene	3,51
trans-linalool oxide	0,05	D-germacrene	0,22
linalool	25,58	γ-cadinene	0,08
hotrienol	0,06	caryophyllene oxide	0,10

HELLENIC AGRICULTURAL ORGANISATION DEMETER Institute of Plant Breeding and Genetic Resources

The Deputy Director

Dr P. Chatzopoulou
Senior Researcher

The Chemist

Eleni Lalidou

Customer :

THARROS HERBS & OILS PC
For the attention of Nancy Ntagobudi<br>1st KM Agias Sofias-provincial road Tirnavos-Larisa<br>40100 TIRNAVOS, GREECE

Sample Nature : ESSENTIAL OIL
Botanical name : MELISSA OFFICINALIS
Common name : MELISSE
Batch number : SAMPLE
Origin : -
Plant part : AERIAL PART
Our reference : Y147

Receipt : 01/07/2019
Analysis date : 09/09/2017
Packaging : Aluminium flask of 5mL
Requested analysis : Classic analysis
Sample storage : 1 year - room temperature

Comments and conclusions :
**BATCH IN COMPLIANCE WITH THE STANDARD<br>OF MELISSA ESSENTIAL OIL**

Report validated by : Daniel Dantin - *Laboratory director* Line Pauset - *Quality manager*

SARL **Pyrenessences Analyses**

Analyse d'huiles essentielles & autres matières premières naturelles

GAS CHROMATOGRAPHY (according to the norm NF ISO 11024)

Analysis conditions :

. GC/MS Agilent 7890 / 5977 – Column : VF WAX (polaire) 60 m * 0.25 mm * 0.25 µm
. GC/FID Agilent 6890 – Column : VF WAX (polaire) 60 m * 0.25 mm * 0.25 µm
. Temperature program : 5 min to 60°C - 3°C/min until 250°C - 5 min to 250°C
. Carrier gas : He (23 psis/MS – 30 psis/FID)
. Sample injection / split : 1 µl of 10 % solution in hexane
. Mass range : 30 to 350 - Oil compounds are identified by a combination of retention times and mass spectra library (our own database and NIST commercial database).
. Percentages are calculated from GC/FID peaks areas without using corrections factors.

Chromatographic profile :

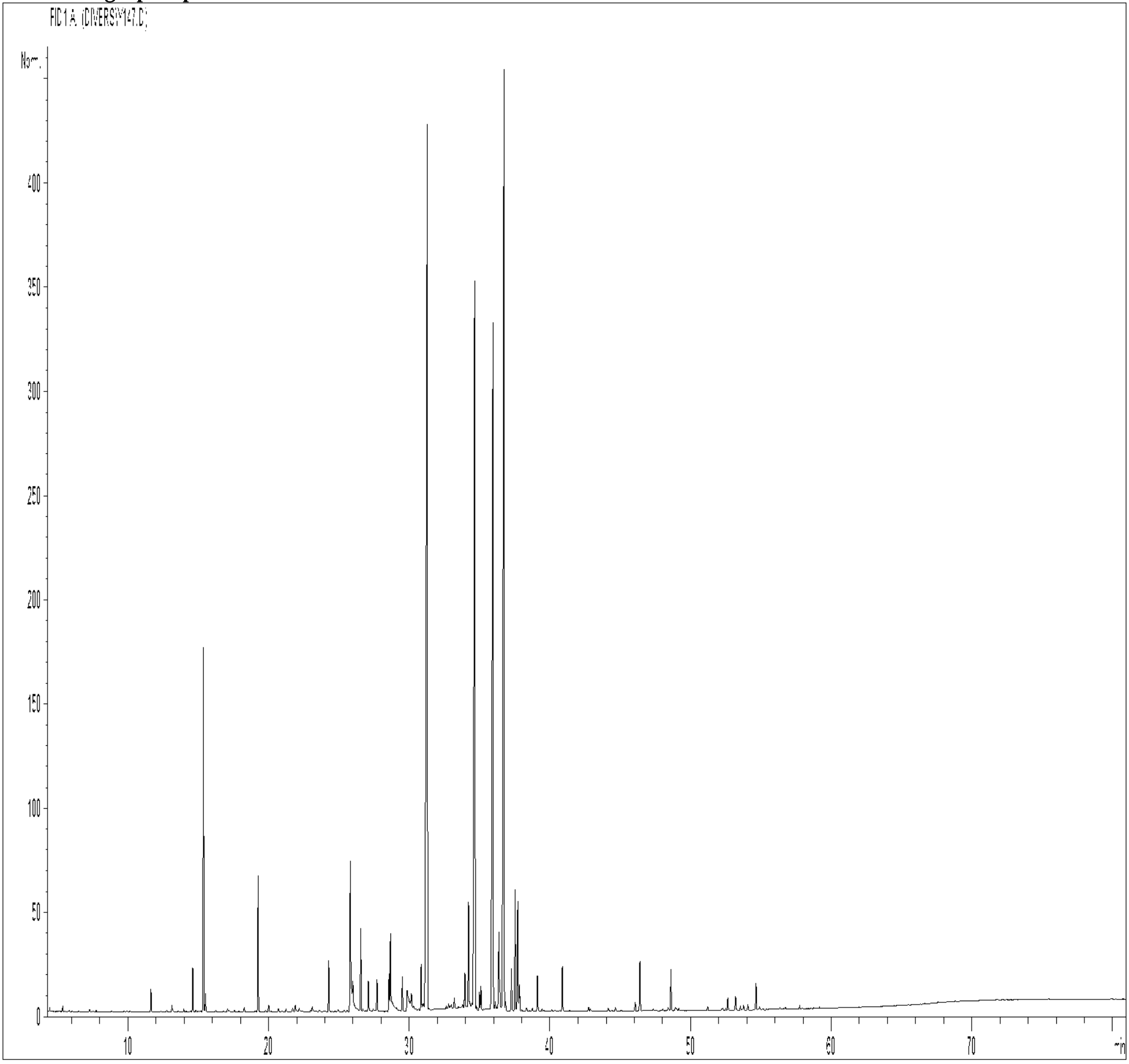

SARL PYRENESSENCES ANALYSES - 2, chemin de la plaine - 11340 Belcaire, FRANCE
TEL: +33 (0)4 68 31 77 83 - Courriel : analyses@pyrenessences.com
SIRET 511 930 869 00017 - Code APE 7490B

Identification results : MELISSA OIL					
Pics	RT (min)	Compound name	%	Norm (%)	Allergens (%)
1	4.44	ACETONE	0.04		
2	5.34	BUTANAL 2-METHYL	0.02		
3	5.38	ISOVALERALDEHYDE	0.03		
4	5.85	FURAN 2-ETHYL	0.02		
5	7.29	alpha-PINENE	0.02		
6	7.75	FURAN.2.5-DIETHYLTETRAHYDRO-	0.02		
7	10.15	SABINENE	0.02		
8	11.65	beta-MYRCENE	0.22		
9	12.77	2,3-DEHYDRO-1,8-CINEOL	0.02		
10	13.14	LIMONENE	0.07		0.07
11	13.18	1-BUTANOL, 3-METHYL-	0.02		
12	13.98	2-HEXENAL	0.03		
13	14.61	Cis-beta-OCIMENE	0.45		
14	14.83	gamma-TERPINENE	0.02		
15	15.39	**Trans-beta-OCIMENE**	**4.17**		
16	15.52	3-OCTANONE	0.18		
17	16.27	HEXYL ACETATE	0.02		
18	17.08	HEXENYLIC ESTER + OCTANAL	0.03		
19	17.55	TRIDECANE	0.02		
20	18.26	3-HEXEN-1-OL ACETATE	0.05		
21	19.03	2-HEXEN-1-OL ACETATE	0.02		
22	19.24	6-METHYL-5-HEPTEN-2-ONE	1.52		
23	19.78	1-HEXANOL	0.02		
24	20.00	Cis-ROSE EPOXIDE + 5-HEPTENAL.2.6-DIMETHYL-	0.08		
25	20.68	Trans-ROSE EPOXIDE	0.03		
26	20.93	ALLO-OCIMENE	0.02		
27	21.22	3-HEXEN-1-OL	0.03		
28	21.71	3-OCTANOL	0.05		
29	21.89	NONANAL	0.11		
30	22.17	GALBANOLENE	0.04		
31	22.21	COMPOUND Mw=150	0.02		
32	22.98	PERILLENE	0.02		
33	23.10	PHOTOCITRAL ISOMER	0.08		
34	23.25	COMPOUND Mw=152	0.02		
35	23.62	LINALOOL cis-OXIDE	0.02		
36	24.27	1-OCTEN-3-OL	0.59		
37	24.93	alpha-CUBEBENE	0.03		
38	25.36	NERYL OXIDE	0.02		
39	25.80	**CITRONELLAL**	**2.48**		
40	25.98	ISOGERANIAL	0.78		
41	26.54	alpha-COPAENE	1.08		
42	26.70	DECANAL	0.05		
43	27.09	ISONERAL	0.41		
44	27.48	alpha-BOURBONENE	0.02		
45	27.70	beta-BOURBONENE	0.44		
46	28.56	beta1-CUBEBENE	0.51		
47	28.66	LINALOOL	1.00		1.00
48	28.80	PHOTOCITRAL A	0.43		
49	29.09	1-OCTANOL	0.10		
50	29.50	METHYL CITRONELLATE	0.46		

Identification results : MELISSA OIL

Pics	RT (min)	Compound name	%	Norm (%)	Allergens (%)
51	29.85	TERPENIC ALCOHOL	0.74		
52	30.14	epsilon-CADINENE	0.37		
53	30.29	SESQUITERPENE	0.12		
54	30.85	beta-ELEMENE	0.63		
55	30.99	beta-CUBEBENE	0.17		
56	31.28	**beta-CARYOPHYLLENE**	**21.55**		
57	32.62	CADINA-3,5-DIENE	0.10		
58	32.79	SESQUITERPENE	0.15		
59	32.91	METHYL NERATE	0.07		
60	32.98	SESQUITERPENE	0.14		
61	33.20	ALLO-AROMADENDRENE	0.26		
62	33.49	CITRONELLYL ACETATE	0.10		
63	33.78	ZONARENE	0.20		
64	33.94	E-beta-FARNESENE	0.62		
65	34.21	alpha-HUMULENE	1.65		
66	34.45	Cis-4,5-MUUROLADIENE	0.16		
67	34.65	**NERAL**	**13.20**		13.20
68	34.77	TERPENIC ALCOHOL	0.10		
69	34.98	gamma-MUUROLENE	0.23		
70	35.08	METHYL GERANATE	0.33		
71	35.95	**GERMACRENE D**	**14.70**		
72	36.12	SESQUITERPENE	0.18		
73	36.37	alpha-MUUROLENE	1.21		
74	36.43	SESQUITERPENE	0.32		
75	36.73	**GERANIAL**	**18.40**		18.40
76	36.84	BICYCLOGERMACRENE	0.16		
77	37.27	alpha-FARNESENE	0.55		
78	37.52	GERANYL ACETATE	1.50		
79	37.71	delta-CADINENE	1.63		
80	37.83	gamma-CADINENE	0.34		
81	38.00	SESQUITERPENE	0.02		
82	38.34	METHYL SALICYLATE	0.05		
83	38.74	CADINA-1,4-DIENE	0.04		
84	39.11	NEROL	0.46		
85	39.43	Cis-ISOGERANIOL	0.04		
86	39.55	Trans-ISOGERANIOL	0.02		
87	40.13	beta-DAMASCENONE	0.02		
88	40.33	2-METHYLENEBORNANE	0.02		
89	40.88	GERANIOL	0.56		0.56
90	42.74	EPI-CUBEBOL	0.06		
91	42.88	AROMATIC COMPOUND	0.03		
92	44.15	HUMULENE EPOXIDE	0.05		
93	44.66	CUBEBOL	0.05		
94	46.06	ISOCARYOPHYLLENE EPOXIDE	0.13		
95	46.42	CARYOPHYLLENE EPOXIDE	0.70		
96	48.04	NEROLIDOL	0.03		
97	48.42	EPOXY-6,7-HUMULENE	0.05		
98	48.61	COMPOUND Mw=222	0.55		
99	48.94	EPI-CUBENOL	0.08		
100	49.18	CUBENOL	0.03		

Identification results : MELISSA OIL					
**Pics**	**RT (min)**	**Compound name**	**%**	**Norm (%)**	**Allergens (%)**
101	51.23	PENTADECANONE TRIMETHYL	0.05		
102	52.66	T-CADINOL	0.16		
103	53.20	alpha-MUUROLOL	0.20		
104	53.56	delta-CADINOL	0.05		
105	53.79	CARVACROL	0.07		
106	54.08	GERANYL-alpha-TERPINENE	0.07		
107	54.67	alpha-CADINOL	0.36		
108	54.91	CITRONELLIC ACID	0.04		
109	56.76	CARYOPHYLLA-3,7-DIEN-6-OL	0.03		
110	57.76	GERANIC ACID	0.05		
111	58.77	SESQUITERPENIC EPOXYDE	0.02		
112	59.17	SESQUITERPENIC EPOXYDE	0.02		
113	67.59	AROMATIC COMPOUND	0.05		
		**TOTAL**	**100.00**		**33.23**

HELLENIC AGRICULTURAL ORGANIZATION - DEMETER
DIRECTORATE GENERAL OF AGRICULTURAL RESEARCH N.AG.RE.F.

INSTITUTE OF PLANT BREEDING AND GENETIC RESOURCES
DEPARTMENT OF AROMATIC AND MEDICINAL PLANTS
P.O.Box 60458, GR 57001, THERMI, THESSALONIKI, GREECE
TEL.: +30 2310 471 110, ext. 0 202
FAX: +30 2310 473 024
Email: chatzopoulou@ipgrb.gr
aromparc.ethiage@gmail.com

Thessaloniki, 14 July 2019
Prot.no: 1698

To THARROS SA – TZIOUMAKIS Bros
5th km Tirnavos-Larisa
GR 40100, TIRNAVOS
GREECE

CERTIFICATE OF ANALYSIS

Sample number	:	120/2019
Sample type	:	OREGANO ESSENTIAL OIL
Costumer	:	THARROS SA – TZIOUMAKIS Bros
Region	:	TIRNAVOS – LARISA, GREECE

ESSENTIAL OIL ANALYSIS

COMPOUND	% YIELD	COMPOUND	% YIELD
α-thujene	0,48	α-terpinolene	0,08
α-pinene	0,47	p-cymenene	0,06
thuja 2,4-(10) diene	0,05	linalool	0,12
camphene	0,13	borneol	0,37
β-pinene	0,09	terpinen-4-ol	0,55
1-octen-3-ol	0,22	carvacrol methyl ether	0,40
β-myrcene	0,78	thymol	4,79
α-phellandrene	0,12	carvacrol	76,60
δ-3-carene	0,04	β-caryophyllene	1,42
α-terpinene	0,78	α-humulene	0,14
p-cymene	7,42	β-bisabolene	1,08
limonene	0,17	γ-cadinene	0,05
β-phellandrene	0,13	δ-cadinene	0,07
γ-terpinene	3,05	caryophyllene oxide	0,18
cis-sabinene hydrate	0,13		

The Deputy Director
Dr P. Chatzopoulou
Senior Researcher

The Chemist
Eleni Lalidou
