# Supplementary material for: Comparative Studies on the Anti-Inflammatory and Apoptotic Activities of Four Greek Essential Oils: Involvement in the Regulation of NF-κΒ and Steroid Receptor Signaling
Source: Life (Basel). 2023 Jul 10;13(7):1534. doi: 10.3390/life13071534 (PMC10381560; doi:10.3390/life13071534)
Supplement: Supplementary file 1 [file life-13-01534-s001.zip › Supplementary Figure S4.pdf]

Comparative Studies on the Anti-Inflammatory and Apoptotic Activities of Four Greek Essential Oils: Involvement in the Regulation of NF-κB and Steroid Receptor Signaling

Figure S4: Representative images used in western blot analysis

Figure 4A. GR-PEPCK films

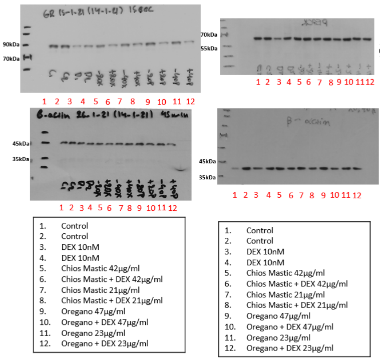

Figure 6A. p65 films

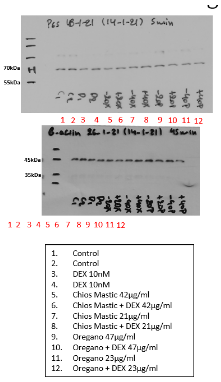

Figure 7A. procasp-3 films

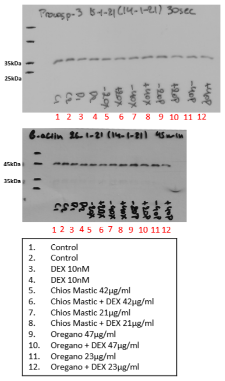

Figure 4A-6A & 7A Procasp-3, GR, p65	Densitometry readings			
	**Procasp-3**	**GR**	**p65**	**β-actin**
**Control**	9304	12758	8594	7800
**Control**	9997	13737	9778	8801
**DEX 10nM**	6670	3015	6343	7421
**DEX 10nM**	7844	6497	8150	8756
**Chios Mastic 42µg/ml**	7204	8759	5466	7655
**Chios Mastic + DEX 42µg/ml**	7301	4359	5640	6045
**Chios Mastic 21µg/ml**	9041	9276	6176	7555
**Chios Mastic + DEX 21µg/ml**	10674	7734	9337	8976
**Oregano 47µg/ml**	10809	13261	9869	12094
**Oregano + DEX 47µg/ml**	7245	5197	5459	9055
**Oregano 23µg/ml**	8940	13698	6264	8807
**Oregano + DEX 23µg/ml**	7457	3993	6803	5900

Figure 4A-6A & 7A Procasp-3, GR, p65	Relative protein levels (protein densitometry readings/β-actin densitometry readings)		
	**Procasp-3**	**GR**	**p65**
**Control**	1.192821	1.635641	1.101795
**Control**	1.135894	1.560845	1.11101
**DEX 10nM**	0.898801	0.406279	0.854737
**DEX 10nM**	0.895843	0.742005	0.93079
**Chios Mastic 42µg/ml**	0.941084	1.144219	0.714043

**Chios Mastic + DEX 42μg/ml**	1.207775	0.721092	0.933002
**Chios Mastic 21μg/ml**	1.196691	1.227796	0.817472
**Chios Mastic + DEX 21μg/ml**	1.189171	0.861631	1.040218
**Oregano 47μg/ml**	0.893749	1.096494	0.816024
**Oregano + DEX 47μg/ml**	0.80011	0.573937	0.602871
**Oregano 23μg/ml**	1.015102	1.555354	0.711252
**Oregano + DEX 23μg/ml**	1.263898	0.67678	1.153051

**Figure 4A-6A & 7A Procasp-3,GR,p65**	**Ratio (Relative sample protein levels/Relative control protein levels)**		
	**Procasp-3**	**GR**	**p65**
**Control**	1	1	1
**Control**	1	1	1
**DEX 10nM**	0.8	0.2	0.8
**DEX 10nM**	0.8	0.5	0.8
**Chios Mastic 42μg/ml**	0.8	0.7	0.6
**Chios Mastic + DEX 42μg/ml**	1	0.4	0.8
**Chios Mastic 21μg/ml**	1	0.8	0.7
**Chios Mastic + DEX 21μg/ml**	1	0.5	0.9
**Oregano 47μg/ml**	0.7	0.7	0.7
**Oregano + DEX 47μg/ml**	0.7	0.4	0.5
**Oregano 23μg/ml**	0.9	1	0.6
**Oregano + DEX 23μg/ml**	1	0.4	1

**PEPCK**	**Densitometry readings**	
	**PEPCK**	**β-actin**
**Control**	8409	1965
**Control**	9875	12202
**DEX 10nM**	5526	7182
**DEX 10nM**	7918	10030
**Chios Mastic 42μg/ml**	8162	10554
**Chios Mastic + DEX 42μg/ml**	8347	8305
**Chios Mastic 21μg/ml**	8182	8801
**Chios Mastic + DEX 21μg/ml**	6996	6474
**Oregano 47μg/ml**	9515	10210
**Oregano + DEX 47μg/ml**	7981	9398
**Oregano 23μg/ml**	8896	9498
**Oregano + DEX 23μg/ml**	9563	6791

**PEPCK Figure 4A**	**Relative protein levels (protein densitometry readings/β-actin densitometry readings)**
	**PEPCK**

**Control**	0.809294
**DEX 10nM**	0.769424
**DEX 10nM**	0.789432
**Chios Mastic 42μg/ml**	0.773356
**Chios Mastic + DEX 42μg/ml**	1.005057
**Chios Mastic 21μg/ml**	0.929667
**Chios Mastic + DEX 21μg/ml**	1.08063
**Oregano 47μg/ml**	0.931929
**Oregano + DEX 47μg/ml**	0.849223
**Oregano 23μg/ml**	0.936618
**Oregano + DEX 23μg/ml**	1.408187

**PEPCK Figure 4A**	**Ratio (Relative sample protein levels/Relative control protein levels)**
	**PEPCK**
**Control**	1
**DEX 10nM**	1
**DEX 10nM**	1
**Chios Mastic 42μg/ml**	1
**Chios Mastic + DEX 42μg/ml**	1.2
**Chios Mastic 21μg/ml**	1
**Chios Mastic + DEX 21μg/ml**	1.3
**Oregano 47μg/ml**	1.2
**Oregano + DEX 47μg/ml**	1
**Oregano 23μg/ml**	1.2
**Oregano + DEX 23μg/ml**	1.7

Figure 4B. GR-PEPCK films

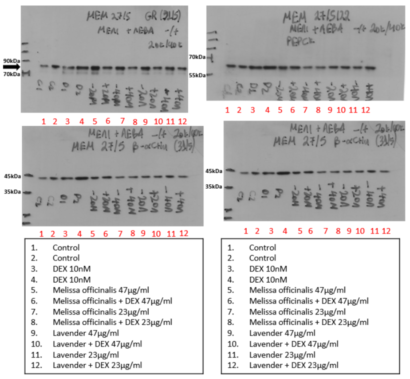

Figure 6B. p65 films

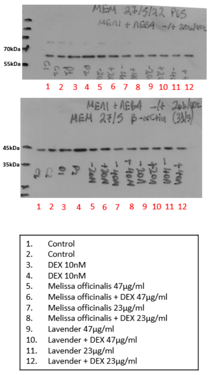

Figure 7B. Procasp-9 & Procasp-3 films

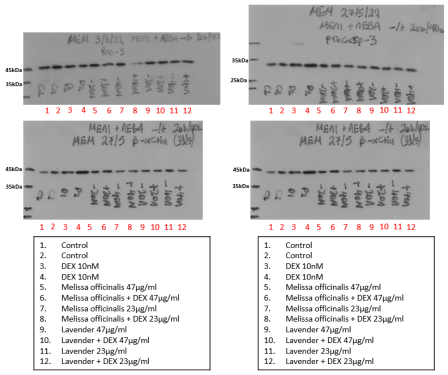

**Figure 4B-6B & 7B<br>GR, PEPCK, Procasp-3, Procasp-9, p65**	**Densitometry readings**					
	**GR**	**PEPCK**	**Procasp-3**	**Procasp-9**	**p65**	**b-actin**
**Control**	8517	7724	6302	10244	7276	6417
**Control**	9835	8680	7136	11523	8177	7584

**DEX 10nM**	7754	14809	8841	12748	11433	10641
**DEX 10nM**	10378	13787	10049	14438	12102	12050
**Melissa Officinalis 47μg/ml**	8318	7540	6153	10950	13683	9647
**Melissa Officinalis + DEX 47μg/ml**	5541	14753	7189	7087	8328	9799
**Melissa Officinalis 23μg/ml**	7112	6804	4489	8581	10942	8389
**Melissa Officinalis + DEX 23μg/ml**	4118	5937	4678	4149	7134	7411
**Lavender 47μg/ml**	7471	6178	6014	8616	5527	7501
**Lavender + DEX 47μg/ml**	5157	8457	6289	9282	5889	6107
**Lavender 23μg/ml**	7392	7414	5966	8944	5127	5894
**Lavender + DEX 23μg/ml**	5598	8512	5781	7686	6684	5619

**Figure 4B-6B & 7B<br>GR, PEPCK, Procasp-3, Procasp-9, p65**	**Relative protein levels (protein densitometry readings/β-actin densitometry readings)**				
	**GR**	**PEPCK**	**Procasp-3**	**Procasp-9**	**P65**
**Control**	1.327256	1.203678	0.982079	1.596385	1.133863
**Control**	1.296809	1.144515	0.940928	1.519383	1.078191
**DEX 10nM**	0.728691	1.391693	0.830843	1.198008	1.074429
**DEX 10nM**	0.861245	1.144149	0.833942	1.198174	1.004315
**Melissa Officinalis 47μg/ml**	0.862237	0.78159	0.637815	1.135068	1.418368
**Melissa Officinalis + DEX 47μg/ml**	0.565466	1.505562	0.733646	0.723237	0.849883
**Melissa Officinalis 23μg/ml**	0.847777	0.811062	0.535105	1.022887	1.304327
**Melissa Officinalis + DEX 23μg/ml**	0.555661	0.801106	0.631224	0.559843	0.962623
**Lavender 47μg/ml**	0.996001	0.823624	0.80176	1.148647	0.736835
**Lavender + DEX 47μg/ml**	0.844441	1.384804	1.029802	1.519895	0.964303
**Lavender 23μg/ml**	1.254157	1.257889	1.012216	1.517475	0.869868
**Lavender + DEX 23μg/ml**	0.996263	1.51486	1.028831	1.367859	1.189536

**Figure 4B-6B & 7B<br>GR, PEPCK, Procasp-3, Procasp-9, p65**	**Ratio (Relative sample protein levels/Relative control protein levels)**				
	**GR**	**PEPCK**	**Procasp-3**	**Procasp-9**	**P65**
**Control**	1	1	1	1	1
**Control**	1	1	1	1	1
**DEX 10nM**	0.5	1.2	0.8	0.8	0.9
**DEX 10nM**	0.6	1	0.8	0.8	0.9
**Melissa Officinalis 47μg/ml**	0.6	0.6	0.6	0.7	1.3
**Melissa Officinalis + DEX 47μg/ml**	0.4	1.3	0.7	0.5	0.7
**Melissa Officinalis 23μg/ml**	0.6	0.7	0.5	0.6	1.2
**Melissa Officinalis + DEX 23μg/ml**	0.4	0.7	0.6	0.4	0.8
**Lavender 47μg/ml**	0.8	0.7	0.8	0.7	0.6
**Lavender + DEX 47μg/ml**	0.6	1.2	1	1	0.9
**Lavender 23μg/ml**	0.9	1	1	1	0.8
**Lavender + DEX 23μg/ml**	0.8	1.3	1	0.9	1
