# Supplementary material for: Comparative Studies on the Anti-Inflammatory and Apoptotic Activities of Four Greek Essential Oils: Involvement in the Regulation of NF-κΒ and Steroid Receptor Signaling
Source: Life (Basel). 2023 Jul 10;13(7):1534. doi: 10.3390/life13071534 (PMC10381560; doi:10.3390/life13071534)
Supplement: Supplementary file 1 [file life-13-01534-s001.zip › Supplementary Figures S1-S3.pdf]

Supplementary Materials

Comparative Studies on the Anti-Inflammatory and Apoptotic Activities of Four Greek Essential Oils: Involvement in the Regulation of NF-κB and Steroid Receptor Signaling

Achilleas Georgantopoulos [1], Athanasios Vougioukas [1], Foteini D. Kalousi [1], Ioannis Tsialtas [1], Anna-Maria G. Psarra [1,*]

[1] Department of Biochemistry and Biotechnology, University of Thessaly, Biopolis, 41500 Larissa, Greece; ageorgant@uth.gr (A.G.); atvougioukas@uth.gr (A.V); fokalous@uth.gr (F.D.K.); tsialtasj@gmail.com (I.T.); ampsarra@uth.gr (A.-M.G.P)

* Correspondence: ampsarra@uth.gr; Tel.: +30-2410-565221

Supplementary Data S1. Chemical composition of the essential oils from Chios Mastic, *Melissa officinalis*, Oregano, and Lavender

Supplementary Figure S1. Effect of essential oils on ERs transcriptional activation

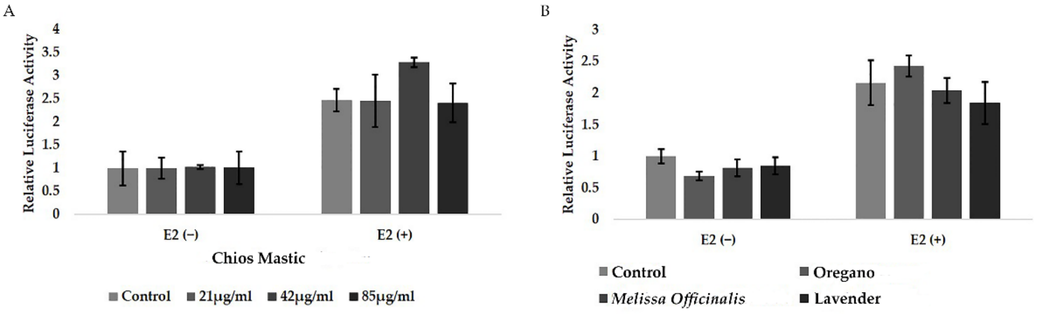

Figure S1. A. Chios Mastic essential oil caused no effect on the ERβ transcriptional activation. Oregano, *Melissa officinalis* and Lavender showed no effect on the ERα transcriptional activation. Cells grown in hormone-free medium for 48 h were transiently co-transfected with a ERE dependent Luciferase reporter gene construct, a PEGFPC2ERβ (A) or PEGFPC2ERα (B) and a β-galactosidase reporter construct. Subsequently, cells were treated with the indicated amounts of the essential oils, in the presence or absence of E2, for 6 h. Then, cells were harvested and lysed. Assessment of the luciferase and β-galactosidase activity was followed in cells extracts. Relative luciferase activity was expressed as normalized against the β-galactosidase activity. Relative luciferase activity in control cells was set as 1. Data were analyzed by two-way ANOVA and are expressed as mean ± SD, (n = 6).

Supplementary Figure S2. Cytotoxic effects of essential oils on HEK293 cells, upon 24h incubation.

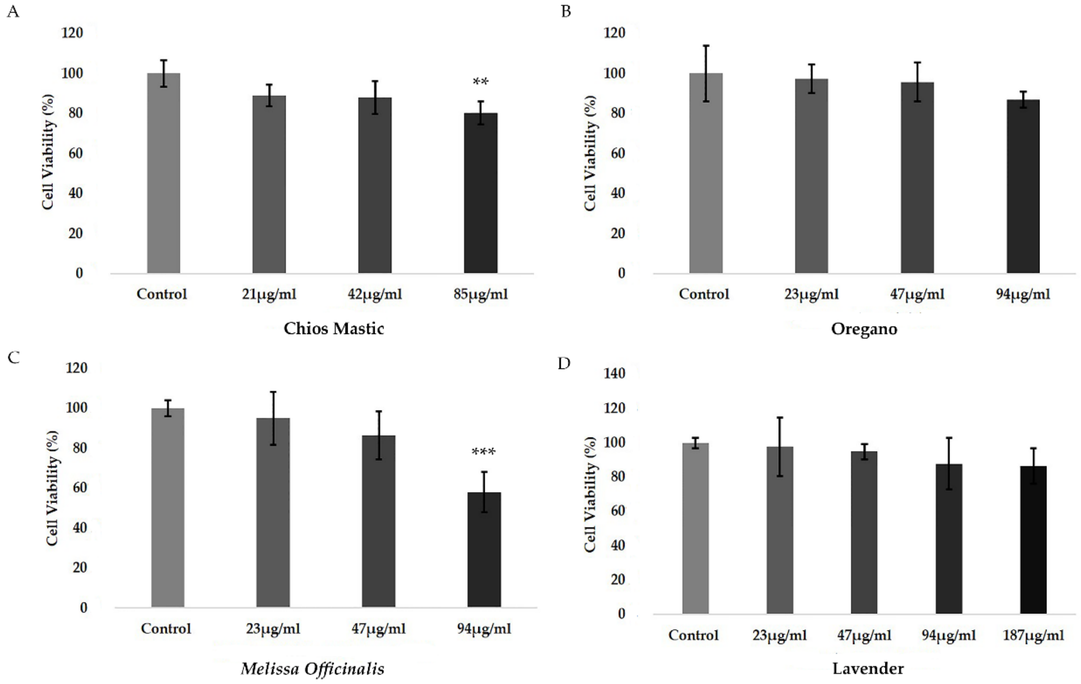

Figure S2. Evaluation of the cytotoxic effects of the essential oils from Chios Mastic, Oregano, *Melissa officinalis* and Lavender on HEK-293 cells. Cytotoxicity was assessed by applying MTT assay in HEK-293 cells subjected to treatment with essential oils for 24h. Viability of control cells was considered as 100%. Relative cell viability (Cell viability, %) is expressed as the viability of cells treated with various

concentrations of the respective essential oil, compared to the viability of the control cells. Data are expressed as mean ± SD (n=5), ** p<0.01, *** p<0.001.

Supplementary Figure S3. Assessment of the anti-inflammatory action of Lavender

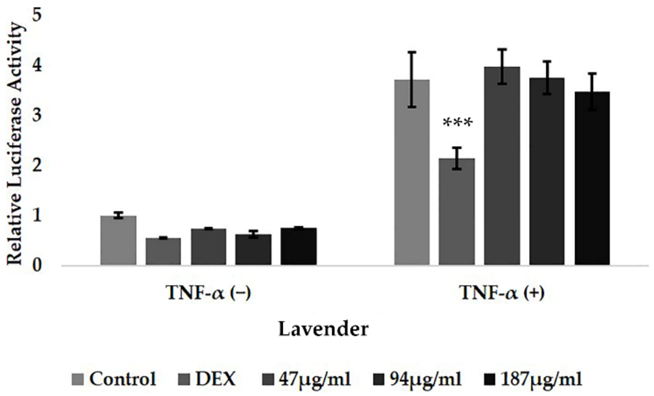

Figure S3. No anti-inflammatory activity was observed by the Lavender essential oil at a concentration range of 47 to 187 μg/ml. HEK-293 cells were co-transfected with the NF-κB reporter luciferase and the β-galactosidase reporter constructs and subsequently were treated with the indicated concentrations of the Lavender essential oil, or with 1μM DEX, for 6h, in the presence or absence of 20ng/ml TNF-*α*, at hormone-free medium. Assessment of the luciferase and the β-galactosidase activity was followed. Results were expressed as relative luciferase activity normalized against β-galactosidase activity. Relative luciferase activity in control vehicle-treated cells was set as 1. Data are expressed as mean ± SD, (*n*=6), *** p<0.001.
